# Learning in Two-Scales Through LSTM-GPT2 Fusion Network: A Hybrid Approach for Time Series Anomaly Detection

**DOI:** 10.3390/s25061849

**Published:** 2025-03-16

**Authors:** Taoyu Wang, Dan Wu, Jun Wang, Jinwei Zhao, Haoming Wang, Dongnan Xie, Hongtao Zhang, Xinhong Hei

**Affiliations:** 1 School of Computer Science and Engineering, Xi’an University of Technology, Xi’an 710048, China; taurielwang@163.com (T.W.); whm1148899269@163.com (H.W.); xdn1363620827@gmail.com (D.X.); zhtttyts@163.com (H.Z.); heixinhong@xaut.edu.cn (X.H.); 2 Xi’an Aerospace Propulsion Institute, Xi’an 710049, China; 18092780145@163.com (D.W.); junw83@163.com (J.W.)

**Keywords:** LSTM, GPT-2, time series data, anomaly detection, feature extraction, clustering, outlier detection

## Abstract

Anomaly detection (AD) in multivariate time series data (MTS) collected by industrial sensors is a crucial undertaking for the damage estimation and damage monitoring of machinery like rocket engines, wind turbine blades, and aircraft turbines. Due to the complex structure of industrial systems and the varying working environments, the collected MTS often contain a significant amount of noise. Current AD studies mostly depend on extracting features from data to obtain the information associated with various working states, and they attempt to detect the abnormal states in the space of the original data. Nevertheless, the latent space, which includes the most essential knowledge learned by the network, is often overlooked. In this paper, a multi-scale feature extraction and data reconstruction deep learning neural network, designated as LGFN, is proposed. It is specifically designed to detect anomalies in MTS in both the original input space and the latent space. In the experimental section, a comparison is made between the proposed AD process and five well-acknowledged AD methods on five public MTS datasets. The outcomes demonstrate that the proposed method attains state-of-the-art or comparable performance. The memory usage experiment illustrates the space efficiency of LGFN in comparison to another AD method based on GPT-2. The ablation studies emphasise the indispensable role of each module in the proposed AD process.

## 1. Introduction

Anomaly detection (AD) is a data mining task that aims to identify unusual or abnormal data within a large volume of standard data. The applications of AD are numerous and diverse. They include the detection of financial fraud, as evidenced by the work of Rousseeuw et al. [1], Sanchez et al. [2], and Badawi et al. [3], as well as the monitoring of machine health in industrial settings, as demonstrated by Qiao et al. [4], de et al. [5], and Yang et al. [6], and the detection of seizures in medical diagnostics [7,8,9]. A significant portion of these real-world systems are online in nature, with multiple sensors being used to monitor them. Each sensor generates a one-dimensional data sequence, which can then be combined and analysed to assess the overall health status of the equipment. This process is known as multivariate time series anomaly detection (MTAD).

Statistical methods for AD include the exponentially weighted moving average (EWMA), isolation forest [10], auto-encoders [11], and other methods [12,13,14,15]. However, when dealing with the vast amounts of MTS generated in complex environments, ***these traditional methods often prove inadequate in terms of efficiency and accuracy due to interference from noisy data.***

Another category of AD that has gained considerable popularity employs recurrent neural networks (RNN) and variational auto-encoders (VAE) [16] to learn temporal dependencies and the distribution of standard data sequences. Due to the unique structure of RNN, this type of network can capture short-term correlation of time series data, so it has been widely studied. The VAE-LSTM hybrid model proposed in [17] combines a VAE to extract local features and an LSTM to capture longer temporal correlations, which allows it to detect anomalies over multiple time scales. Another RNN-based method is the LSTM-based VAE-GAN [18], which combines LSTMs with a VAE and a generative adversarial network (GAN) framework to jointly train the encoder, generator, and discriminator, effectively reducing mapping errors and improving AD accuracy and speed. Notwithstanding their advantages, ***these methods frequently encounter difficulties in capturing long-term dependency.*** Furthermore, VAEs are prone to difficulties such as KL diverge vanishing and over-regulation.

The advent of transformers [19] has provided new opportunities in the AD field by enabling the exploration of correlations between distant data points using the attention mechanism. The first research that utilized a transformer to detect anomalies in time series data was Anomaly Transformer [20]. It utilizes a stacking structure of alternating anomaly-attention blocks and feed-forward layers, with residual connections and layer normalization, to learn deep multi-level features and underlying association data. The MT-RVAE proposed in [21] addresses the limitations of traditional transformers by incorporating global context in addition to local information for sequential analysis. The transformer model overcomes the inability of RNNs to perform parallel computations, and the number of operations required by the transformer model to compute correlations between two sequences does not increase with distance. Research on AD algorithms based on transformers has become a hot topic in recent years. However, calculating attention scores involves matrix multiplications, ***resulting in a quadratic increase in temporal and spatial complexity as the length of the time series grows.*** This is an inherent limitation for all transformer-based AD methods [22].

Large language models (LLMs) have garnered significant attention in recent years due to their remarkable ability to understand and generate human-like text. The foundation of LLMs lies in the transformer architecture, which replaces traditional recurrent and convolutional neural networks (CNNs) with self-attention mechanisms. This architecture enables models to capture long-range dependencies and contextual relationships within text, making them highly effective for natural language processing (NLP) tasks. The evolution of LLMs culminated in the development of autoregressive models like GPT. The GPT series, developed by OpenAI, has been instrumental in advancing the capabilities of LLMs. GPT-1 [23] was a proof-of-concept model demonstrating the effectiveness of pre-training on vast amounts of text data followed by fine-tuning on specific tasks. GPT-2 [24] extends this by increasing the model size and the amount of training data, leading to significant improvements in text generation quality and fluency.

Since LLM was originally designed for NLP tasks, applying it to MTS-related tasks inevitably requires leveraging knowledge transfer techniques. Upon investigation, research that applies LLM to MTAD tasks is scarce, and many questions can be explored in this direction. Ref. [25] demonstrates that the LLM-based monitor can effectively detect semantic anomalies in a way that shows agreement with human reasoning. This work extends the application of LLM to recognize semantic anomalies, showing that LLM can be used in the AD domain, ***but does not yet apply LLM to detecting anomalies in time series data.*** Ref. [26] provides a detailed explanation of methods for applying GPT-2 to downstream tasks such as time-series classification, AD, imputation, long/short-term forecasting, and few/zero-shot forecasting. However, ***deploying large models in practical engineering applications is challenging due to their enormous number of parameters.*** In this paper, the value of LLM in the MTAD field is explored while working on solving the problem of deploying large models on small devices.

One characteristic of anomalous events is their rarity. This leads to a scarcity of labels, or category imbalance. Thus, unsupervised AD methods have emerged as a prominent area of research. The goal of unsupervised tasks is to extract critical information. Consequently, MTAD based on machine learning (ML) or deep learning (DL) typically involves a feature extraction network. In most of the time series feature extraction research, like [27,28,29,30], the unit for extraction is a time window, and one window corresponds to one feature. If short time windows are adopted, the extracted features are fine-grained. Due to the length of the time series being fixed, the network will have a large number of features to deal with, leading to issues such as insufficient memory, prolonged training time, poor real-time detection, and challenges in small device deployment. Furthermore, fine-grained features may not be condensed enough. They may contain a significant amount of noise not related to anomalies. Conversely, the extracted features are coarse-grained if long time windows are used, which may result in losing key detail information. Therefore, ***balancing the coarseness and fineness of feature granularity, finding a way to compensate for the loss of detailed information in coarse-grained features, and modelling cross-window dependencies*** are all important topics in time series feature extraction research.

Most existing MTAD approaches use the reconstruction error directly as an anomaly score to detect anomalies in the original input space. However, ***the extracted features that represent the core information are largely overlooked.*** To the best of our knowledge, there is limited research on detecting anomalies in both input spaces and latent spaces.

To address the challenges mentioned above, a novel DL network called LGFN (LSTM-GPT2 Fusion Network) is proposed. This hybrid model has two main functions: multi-scale feature extraction and data reconstruction. The extracted features and reconstructed data are then utilized to detect anomalies in both the input space and the latent space. The main contributions of this work are as follows:

1. The proposed LGFN is a novel approach that combines the advantages of bidirectional LSTM (Bi-LSTM) and generative pre-trained transformer (GPT). It is capable of extracting features of MST at different scales and granularities.
2. Clustering in the latent space is conducted to unravel hidden data patterns. The resulting classification outcomes may be associated with disparate information in various windows and could potentially indicate anomalies in the original space. The outlier score is defined to quantify the extent of deviation.
3. An enhanced anomaly score was devised based on the reconstruction score and outlier score, which are used to detect anomalies in two distinct spaces.

A series of experiments was conducted to evaluate the effectiveness and efficiency of the proposed model. First, the results of the comparative experiment demonstrated that LGFN exhibits SOTA or comparable performance to the prevailing AD methods on a range of datasets. Second, the assessment of memory usage demonstrated the superior spatial efficiency of the proposed method. Finally, the results of the ablation experiment demonstrated the efficacy of each component of the proposed AD process.

The remainder of this paper is structured as follows: Section 2 provides a comprehensive account of LGFN and the methodology employed in utilizing this network for AD. Section 3 presents the results of the experiments, including the settings, the datasets, the results, and a detailed analysis. In conclusion, Section 4 presents suggestions for future research.

## 2. LSTM-GPT2 Fusion Network

In this paper, LGFN is proposed to perform MTAD. The methodology encompasses multi-scale feature extraction, data reconstruction, and latent space clustering.

**Figure 1:**
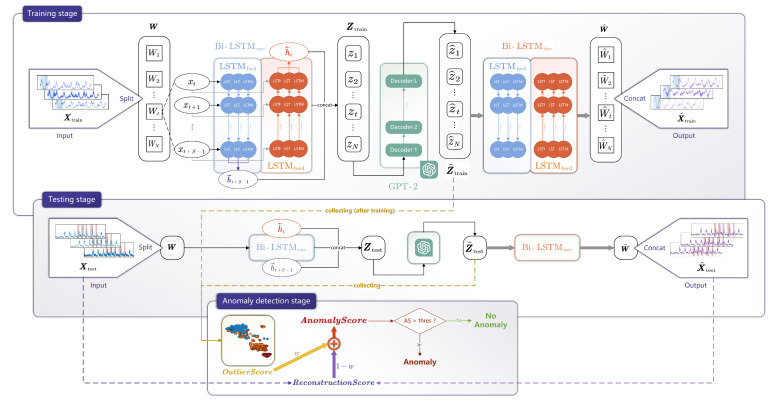
LGFN architecture.

As demonstrated in Figure 1, LGFN comprises three primary modules: a Bi-LSTM encoder, denoted as ${\mathrm{Bi}-\mathrm{LSTM}}_{\mathrm{enc}}$; a decoder with an analogous structure to the encoder, denoted as ${\mathrm{Bi}-\mathrm{LSTM}}_{\mathrm{dec}}$; and a GPT-2. Initially, ${\mathrm{Bi}-\mathrm{LSTM}}_{\mathrm{enc}}$ is employed to extract ***window-level features***, constituting the first stage of feature extraction. Subsequently, the GPT-2 functions as the secondary stage of feature extraction, responsible for the extraction of ***batch-level features***. The process involves the processing of a batch of features with consecutive windows, with the aim of adding long-term dependencies to them. Ultimately, ${\mathrm{Bi}-\mathrm{LSTM}}_{\mathrm{dec}}$ is employed to reconstruct the original data.

LGFN is initially trained on standard data to identify normal patterns; subsequently, it is tested on data that contain anomalies. As LGFN is not trained to recognize abnormal data; anomalous features manifest as outliers during the clustering-based latent space pattern mining process, and the corresponding reconstructed data exhibit significant discrepancies compared to the original. By leveraging the distinct expressions of anomalous data in both spaces, an enhanced anomaly score has been devised, enabling the effective identification of anomalies. This chapter will provide a comprehensive overview of LGFN, including its architecture, training methodologies, and application in AD.

### 2.1. Problem Statement

In general, an MTS dataset can be described as $X=[x_{1},x_{2},\ldots ,x_{t},\ldots ,x_{T}]$, where $x_{t}=\left(x_{t}^{1},x_{t}^{2},\ldots ,x_{t}^{M}\right)$ is an *M*-dimensional reading at the *i*-th time stamp that contains information about *M* different channels. The entire sequence X is further split into several non-overlapping windows $W=\{W_{1},W_{2},\ldots ,W_{t},\ldots ,W_{N}\}$, where $W_{t}=\left[x_{t},x_{t+1},\ldots ,x_{t+S-1}\right]$ includes the future *S* readings counting forward from time stamp *t*. The purpose is to predict a point-wise binary output ${\hat{y}}_{t}\in \left\{0,1\right\}$ for each time stamp *t* with 1 indicating the reading at *t* is an anomaly.

### 2.2. A New Multi-Scale Feature Extraction Structure for MTS

In this section, the way ${\mathrm{Bi}-\mathrm{LSTM}}_{\mathrm{enc}}$ and GPT-2 together perform multi-scale feature extraction to make LGFN a fast and accurate AD network is explained in detail.

In the data processing stage, X is split into small windows with length *S*, yielding an input window sequence $W=\{W_{1},W_{2},\ldots ,W_{N}\}$. ${\mathrm{Bi}-\mathrm{LSTM}}_{\mathrm{enc}}$ transforms $W\in R^{N\times S\times M}$ into a sequence of latent variables $Z=\left\{z_{1},z_{2},\ldots ,z_{N}\right\}\in R^{N\times e}$. Each z∈Z is a feature of dimension *e*. The transformation process is detailed in Equations (1) to (3).

In the context of a specified window, denoted by $W_{t}=\left[x_{t},x_{t+1},\ldots ,x_{t+S-1}\right]$ , the ${\mathrm{Bi}-\mathrm{LSTM}}_{\mathrm{enc}}$ is defined as follows: The forward part ${\mathrm{LSTM}}_{\mathrm{fwd}}$ computes the forward hidden state ${\vec{h}}_{i}$ for each time point (also known as reading) $x_{i}\in R^{M}$ using Equation (1); similarly, the backward part ${\mathrm{LSTM}}_{\mathrm{bwd}}$ computes the backward hidden state ${\overset{\leftarrow }{h}}_{i}$ using Equation (2).

(1) $$\begin{array}{ccc} {\vec{h}}_{t}=0,\;{\vec{h}}_{i} & ={\mathrm{LSTM}}_{\mathrm{fwd}}\left(x_{i},{\vec{h}}_{i-1}\right),\; & \mathrm{for}\;i=t+1,t+2,\ldots ,t+S-1, \end{array}$$

(2) $$\begin{array}{ccc} {\overset{\leftarrow }{h}}_{t+S-1}=0,\;{\overset{\leftarrow }{h}}_{i} & ={\mathrm{LSTM}}_{\mathrm{bwd}}\left(x_{i},{\overset{\leftarrow }{h}}_{i+1}\right),\; & \mathrm{for}\;i=t+S-2,t+S-3,\ldots ,t. \end{array}$$

Then, the forward and backward hidden states are concatenated to form a feature:

(3) $$\begin{array}{cc} z_{t} & =\mathrm{concat}\left({\vec{h}}_{t+S-1},{\overset{\leftarrow }{h}}_{t}\right),\;z_{t}\in R^{e}, \end{array}$$

(4) $$\begin{array}{cc} Z & =\{z_{1},z_{2},\ldots ,z_{t},\ldots ,z_{N}\}. \end{array}$$

Here, $N=\lfloor \frac{T}{S}\rfloor$ is the number of windows.

If the original input data are reconstructed using Z alone, as is customary in most AD networks, then the long-term relationships between windows are likely to be overlooked, restricting the time range considered to only *S* readings. However, since Z represents features from adjacent windows, it is reasonable to hypothesize that these features exhibit temporal dependencies as well. To address this, GPT-2 is introduced as a batch-level feature extractor to sequentially process these features, enabling the management of long-term dependencies in extensive time series data.

**Figure 2:**
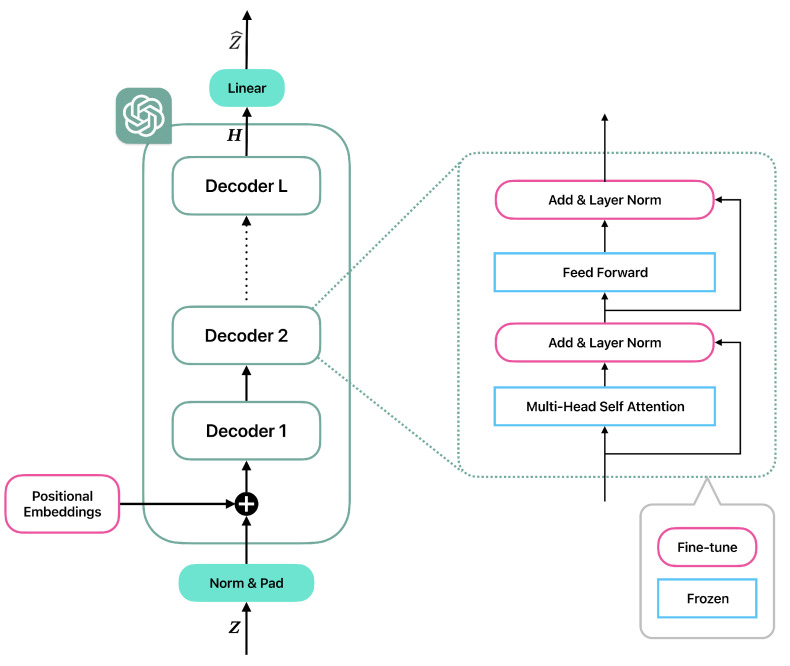
The detailed structure of GPT-2.

Given that GPT-2 was originally developed for the NLP domain, with the objective of adapting its pre-trained parameters for MTAD tasks, its multi-head self-attention layers and feed-forward neural networks are chosen to be frozen, thereby preserving prior knowledge. Furthermore, only the positional embeddings layer and normalization layers are fine tuned, in accordance with the approach outlined in [26]. The detailed structure of GPT-2 is shown in Figure 2.

Before extracting batch-level features, padding is applied to adjust the dimension of Z, ensuring that it aligns with the lightweight GPT-2 model’s typical input requirement. Subsequently, $Z_{\mathrm{pad}}$ is fed into GPT-2, and only the last hidden state is drawn as $\hat{Z}$, which serves as features enriched with temporal information within a batch of *N* windows. These two steps are illustrated in Equation (5) and (6).

(5) $$\begin{array}{cc} \; & Z_{\mathrm{pad}}=\mathrm{Pad}\left(Z,768-e\right), \end{array}$$

(6) $$\begin{array}{cc} \; & \hat{Z}={\mathrm{GPT}-2}_{\mathrm{last}}\;\mathrm{hidden}\left(Z_{\mathrm{pad}}\right). \end{array}$$

${\mathrm{Bi}-\mathrm{LSTM}}_{\mathrm{enc}}$ compresses a window of length *S* into a single feature z. While GPT-2 processes the *N* features in Z, it in fact processes N×S readings in the original input sequence X. This strategy significantly reduces the time and space complexity of the GPT-2 inside LGFN. The subsequent subsections provide detailed analyses and experiments related to space usage.

In Figure 2, the GPT-2 used in LGFN consists of *L* transformer decoders, all of which contain a Multi-Head Self Attention layer, an Add & Layer Norm layer, a Feed Forward layer and another Add & Layer Norm layer. Before the first decoder, the Positional Embeddings layer is used to add temporal information of a bath of features. Layers indicated by blue right-angled rectangles remain unchanged during co-training with Bi-LSTM encoder-decoder architecture, and layers indicated by pink rounded rectangles are fine tuned.

### 2.3. Clustering-Based Latent Space Pattern Mining

In order to identify latent patterns associated with the status of MTS and enhance the interpretability of anomalies, a clustering procedure is employed on standard data. The deviation degree, denoted by $dd_{j}$, is calculated for each $z_{j}$ in ${\hat{Z}}_{\mathrm{test}}\in R^{N_{\mathrm{test}}\times e}$, where $N_{\mathrm{test}}$ is the number of features in the test set. Pattern mining and deviation degree calculation are conducted using the following steps:

1. **Clustering.** Perform a clustering algorithm on ${\hat{Z}}_{\mathrm{train}}$, obtaining $n_{c}$ clusters and their centres $C=\left\{c_{1},c_{2},\ldots ,c_{n_{c}}\right\}\subseteq {\hat{Z}}_{\mathrm{train}}$;
2. **Latent variables distribution.** For each $z_{j}\in {\hat{Z}}_{\mathrm{test}},j=1,2,\ldots ,N_{\mathrm{test}}$, compute its Euclidean distance to all $c_{k}\in C$, and assign it to the nearest cluster. $l_{j}$ is the cluster label of $z_{j}$;
3. **Deviation degree computation.** Define $\mathrm{dist}(z_{j},c_{l_{j}})$ as the Euclidean distance between $z_{j}$ and $c_{l_{j}}$; then, the deviation degree $dd_{j}$ can be obtained using the following definition:
  (7) $$dd_{j}=\mathrm{dist}\left(z_{j},c_{l_{j}}\right)={∥z_{j}-c_{l_{j}}∥}_{2},$$
  the deviation degree vector $\mathrm{Dd}=\{dd_{1},dd_{2},\ldots ,dd_{N_{\mathrm{test}}}\}$.

The detailed calculation steps are illustrated in Algorithm 1.

The subsequent discussion will elucidate the merits of computing deviation degrees based on clustering standard data. In instances where data comprising consecutive anomalous windows are subjected to direct clustering, the anomalous features are likely to coalesce into a small outlier cluster, with a corresponding centre that is also an outlier. Consequently, if the deviation degree is determined by the distance between an outlier feature and its similarly outlying centre, this value may not exceed the deviation degree of normal features. Consequently, such anomalous features may not be detected in the latent space. This underscores the necessity of the proposed Algorithm 1. The algorithm first clusters standard data and then assigns test data to the nearest cluster in order to effectively measure deviation.
**Algorithm 1** Pattern mining and deviation degree calculation1:**Input:** ${\hat{Z}}_{\mathrm{train}}\in R^{N_{\mathrm{train}}\times e}$, ${\hat{Z}}_{\mathrm{test}}\in R^{N_{\mathrm{test}}\times e}$2:**Output:** Deviation degree vector Dd3:Initialize: $N_{\mathrm{train}}=\left\lfloor \frac{T_{\mathrm{train}}}{S}\right\rfloor$, $N_{\mathrm{test}}=\left\lfloor \frac{T_{\mathrm{test}}}{S}\right\rfloor$, $n_{c}$ (number of clusters)4:/* Step 1: Clustering */5:/* Perform clustering on ${\hat{Z}}_{\mathrm{train}}$,6:   obtaining $n_{c}$ clusters and their centres $\mathrm{centres}=\left\{c_{1},c_{2},\ldots ,c_{n_{c}}\right\}\subseteq {\hat{Z}}_{\mathrm{train}}$ */7:$\mathrm{centres}=\mathrm{Clustering}({\hat{Z}}_{\mathrm{train}},n_{c})$8:/* Step 2: Latent variables distribution */9:labels={}10:**for** j=1 to $N_{\mathrm{test}}$ **do**11:    **for**  k=1 to $n_{c}$ **do**12:        $d_{jk}=\mathrm{dist}\left(z_{j},c_{k}\right)$13:    **end for**14:    $l_{j}=\mathrm{labels}\left[z_{j}\right]=\arg\min_{i}d_{ji}$15:    $\mathrm{labels}\leftarrow \mathrm{labels}\cup \{l_{j}\}$16:**end for**17:/* Step 3: Deviation degree computation */18:Dd={}19:**for** j=1 to $N_{\mathrm{test}}$ **do**20:    $dd_{j}=\mathrm{dist}\left(z_{j},c_{l_{j}}\right)$21:    $\mathrm{Dd}\leftarrow \mathrm{Dd}\cup \{dd_{j}\}$22:**end for**23:**Output:** $\mathrm{Dd}=\{dd_{1},dd_{2},\ldots ,dd_{N_{\mathrm{test}}}\}$

### 2.4. Data Reconstruction

The data reconstruction module incorporates a Bi-LSTM window decoder (${\mathrm{Bi}-\mathrm{LSTM}}_{\mathrm{dec}}$) that mirrors ${\mathrm{Bi}-\mathrm{LSTM}}_{\mathrm{enc}}$ and incorporates a fully connected layer fc to adjust the model’s output size, as shown in Equation (8).

(8) $$\hat{X}=W_{fc}\times {\mathrm{Bi}-\mathrm{LSTM}}_{\mathrm{dec}}\left(\hat{Z}\right)+b_{fc},$$

Here, $W_{fc}$ is the weight matrix, and $b_{fc}$ is the bias vector of fc.

## 3. Anomaly Detection Using LGFN

As shown in Figure 1, AD using LGFN incorporates three stages: training, testing, and detecting. The three stages are described in the following subsections.

### 3.1. Training Stage

In the forward pass during training, $X_{\mathrm{train}}$, which includes only normal data, first passes through ${\mathrm{Bi}-\mathrm{LSTM}}_{\mathrm{enc}}$ to obtain Z (Equation (2) to (3)). Then, padding is applied to Z, resulting in $Z_{\mathrm{pad}}$, which converts the encoder’s output into a 768-dimensional vector, so that it meets the input requirements of the pre-trained GPT-2 (Equation (5)). Next, $Z_{\mathrm{pad}}$ is fed into GPT-2, producing features $\hat{Z}$ enriched with long-term temporal relationships (Equation (6)). Finally, ${\mathrm{Bi}-\mathrm{LSTM}}_{\mathrm{dec}}$ and fc perform data reconstruction to obtain ${\hat{X}}_{\mathrm{train}}$ (Equation (8)).

After the forward pass, a standard MSE (mean square error) loss, as described in Equation (9), is back-propagated through ${\mathrm{Bi}-\mathrm{LSTM}}_{\mathrm{dec}}$, GPT-2, and ${\mathrm{Bi}-\mathrm{LSTM}}_{\mathrm{enc}}$ and updates the parameters of these three components together.

(9) $$loss_{\mathrm{MSE}}=\frac{1}{T_{\mathrm{train}}}\frac{1}{M}\sum_{t=1}^{T_{\mathrm{train}}}\sum_{i=1}^{M}{\left(x_{t}^{i}-\hat{x_{t}^{i}}\right)}^{2}.$$

However, inside GPT-2, only the positional embeddings layer and normalization layers are fine tuned, and the multi-head self-attention layers and feed-forward neural networks are chosen to be frozen. This is because GPT-2 learns many general features of sequential data during pre-training. Freezing the multi-head self-attention layers and feed-forward neural networks helps retain these general feature learning capabilities, thus preventing catastrophic forgetting [26]. By fine-tuning the positional embeddings and normalization layers, the model can adapt to the structure and distribution of a specific time series dataset, handling unique time intervals or periodicities more accurately while not altering its understanding of general sequential data patterns. The effectiveness of pre-training is further demonstrated in the ablation experiment in Section 4.4. The pre-trained GPT-2 used in LGFN can be directly downloaded from [31].

As the number of training epochs increases, the loss gradually decreases, ${\mathrm{Bi}-\mathrm{LSTM}}_{\mathrm{enc}}$ learns the features of the time windows, GPT-2 incorporates long-term temporal relationships into the time window features, and finally, the ${\mathrm{Bi}-\mathrm{LSTM}}_{\mathrm{dec}}$ performs precise reconstruction.

After training, the trained LGFN model is saved, and a set of standard features ${\hat{Z}}_{\mathrm{train}}$ from the training set is collected, serving as the known normal operational state.

### 3.2. Testing Stage

In the testing stage, the test set $X_{\mathrm{test}}$, which contains anomalies, is passed through the trained LGFN, yielding test set features ${\hat{Z}}_{\mathrm{test}}$ and reconstruction ${\hat{X}}_{\mathrm{test}}$.

### 3.3. Detecting Stage

The detecting stage is based on ${\hat{Z}}_{\mathrm{train}}$, saved in the training stage, as well as $X_{\mathrm{test}}$, ${\hat{X}}_{\mathrm{test}}$, and ${\hat{Z}}_{\mathrm{test}}$, saved in the testing stage. These components are collected in order to calculate the detection criterion, that is, the anomaly score (AS). The calculation method for (AS) will be explained in detail below.

AS in Equation (12) is based on the reconstruction score (RS), as outlined in Equation (10), and the outlier score (OS), as specified in Equation (11):

(10) $$\begin{array}{cc} \; & \mathrm{RS}=\mathrm{Scaler}(∥X_{\mathrm{test}}-{\hat{X}}_{\mathrm{text}}{∥}_{2}), \end{array}$$

(11) $$\begin{array}{cc} \; & \mathrm{OS}=\mathrm{Scaler}(\mathrm{Smooth}\left(\mathrm{Dd}⊗1_{S}\right)), \end{array}$$

(12) $$\begin{array}{cc} \; & \mathrm{AS}=(1-\omega )\times \mathrm{RS}+\omega \times \mathrm{OS}. \end{array}$$

In Equation (11), Dd is the output obtained by inputting ${\hat{Z}}_{\mathrm{train}}$ and ${\hat{Z}}_{\mathrm{test}}$ into Algorithm 1. ⊗ denotes the Kronecker product; $1_{S}=\left\{1,1,\ldots ,1\right\}$, a vector of length *S*, is used to replicate each element in Dd for *S* times.

(13) $$\mathrm{Dd}⊗1_{S}=\left\{dd_{1}1_{S},dd_{2}1_{S},\ldots ,dd_{N_{\mathrm{test}}}1_{S}\right\}.$$

To improve the continuity of OS across different windows, EWMA is applied as the smoothing function Smooth() in Equation (11). Before combining RS and OS, a scaler is applied to normalize them, ensuring comparability across metrics on the same scale. In Equation (12), the hyper-parameter ω∈[0,1] controls the relative contribution of RS and OS in the final calculation of AS.

To evaluate the basic AD capability of LGFN, a threshold θ is set for AS using the percentile method. Any time point *t* where the anomaly value AS(t)>θ is marked as an anomaly, i.e., let ${\hat{y}}_{t}=1$, otherwise ${\hat{y}}_{t}=0$. The detection result of $X_{\mathrm{test}}$ is ${\hat{Y}}_{\mathrm{test}}=\left\{{\hat{y}}_{1},{\hat{y}}_{2},\ldots ,{\hat{y}}_{T_{\mathrm{test}}}\right\}$.

## 4. Experiments

A series of experiments were conducted under the setup outlined in Table 1 to comprehensively assess the efficacy of LGFN in MTAD tasks.

### 4.1. Baseline Methods and Datasets

#### 4.1.1. Baseline Methods

In order to conduct a comparative experiment, a selection of baseline methods was made consisting of different types of time series AD methods. These included GPT4TS [26], CNN-based TimesNet [32], transformer-based Autoformer [33], and Anomaly Transformer [20], as well as a traditional method: LSTM-VAE [34]. They all performed AD through reconstruction differences.

#### 4.1.2. Datasets

**Figure 3:**
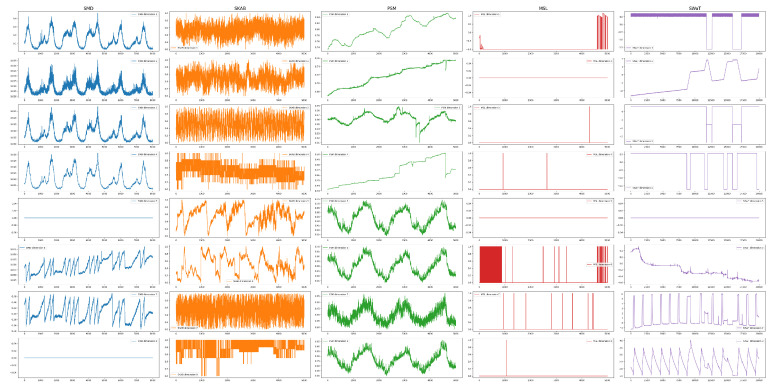
Examples of the five datasets. Due to space limitations, only the first eight dimensions are shown.

In order to demonstrate the efficacy of the proposed AD method, experiments were conducted on five publicly available datasets. These time sequences originated from diverse real-world scenarios, encompassing internet servers (SMD), industrial sensors (SKAB), the public service domain (PSM), the space exploration field (MSL), and industrial control systems (SWaT). The detailed information related to these data sets is described as follows, and the statistics and examples of these datasets are displayed in Table 2 and Figure 3:

1. SMD (Server Machine Dataset) [35] is an MTS dataset collected from server infrastructure, commonly used for benchmarking AD models. It includes labelled anomalies across various server metrics. Its anomaly ratio is only 4.16%, which indicates a category-imbalanced dataset;
2. SKAB (Skoltech Anomaly Benchmark) [36] is a small-scale dataset designed for evaluating AD algorithms. It contains time series data from various industrial and environmental sensors, including both normal samples and anomalies;
3. PSM (Public Service Message) [37] consists of multiple time series instances collected from various public service domains, and each time series captures diverse patterns and temporal dynamics. It includes training sets that only contain normal data and test sets with anomalies;
4. MSL (Mars Science Laboratory rover) [38] is collected from NASA’s Curiosity rover, which is used to explore Mars. The dataset includes telemetry data from various sensors on the rover, such as temperature, voltage, and motor currents. It contains labelled anomalies that simulate potential faults or unusual behaviour in space missions;
5. SWaT (Secure Water Treatment) [39] is a cybersecurity-focused dataset from a scaled-down water treatment plant designed to simulate real-world industrial control systems. It contains MTS with labelled cyber-attacks, making it a valuable resource for research on AD, system security, and resilience in industrial control systems.

Here are some explanations about all five of the datasets we used in this research:

- **Training/test set split.** In Table 2, the training set size $T_{\mathrm{train}}$ and test set size $T_{\mathrm{test}}$ are pre-defined, which is the same as the setting used in [26,32].
- **Handling of missing values.** Some datasets, including PSM, contain values that are NaN (not a number). The occurrence of this issue is related to incomplete data collection or errors during data acquisition. To minimize the impact of these discontinuous data on the following experiments, these values are modified to 0 during preprocessing. Additionally, the labels of all missing data in the test are set to 0.
- **Dataset normalization.** Each dimension of the MTS dataset is assumed to contribute equally to the anomalies. All datasets are normalized using StandardScaler in Equation (14) before being fed into the LGFN model, to eliminate dimensional differences between features and prevent certain features with larger value ranges from having an undue influence on model training.
  (14) $${\bar{x}}_{i}=\frac{x_{i}-\mu_{i}}{\sigma_{i}},\mathrm{for}\;i=1,2,\ldots ,M,$$
  In the above equation, $\mu_{i}$ and $\sigma_{i}$ are the mean and standard deviation of dimension *i*, respectively. The normalized dataset is $X=\{{\bar{x}}_{1},{\bar{x}}_{2},\ldots ,{\bar{x}}_{M}\}$.

Moreover, it is imperative to note that k-medoids clustering has been selected as the pattern mining method in Algorithm 1 to assess the AD performance of LGFN.

### 4.2. Method Comparison

A series of comparative experiments were conducted on the aforementioned five datasets and methods. By comparing the model prediction ${\hat{Y}}_{\mathrm{test}}$ with the ground truth $Y_{\mathrm{test}}=\left\{y_{1},y_{2},\ldots ,y_{T_{\mathrm{test}}}\right\}$, various metrics, such as precision, recall, F1-score and accuracy, can be obtained to evaluate AD performance. It is important to note that AD frequently operates on imbalanced datasets, where anomalies are infrequent relative to normal instances. Consequently, focusing on only precision or recall can generate misleading conclusions. The F1-score provides a more comprehensive view by considering both FPs and FNs, rendering it especially useful when the cost of missing anomalies is as high as incorrectly flagging normal points. Consequently, F1-score is particularly well-suited for evaluating the effectiveness of AD models.

In order to ensure a fair comparison, the embedding dimensions and the length of time series processed per batch were set to be the same for each AD method, with other parameters being optimised as much as possible within the experimental setup. Following the collection of information, it was determined that a 6-layer GPT-2 strikes a good balance compared to using either more or fewer layers, and freezing the model particularly helps prevent catastrophic forgetting, allowing for fine-tuning without the risk of over-fitting. To assess the efficacy of these AD methods under constrained computational resources, a reduced 2-layer GPT-2 configuration was employed for the evaluation of both LGFN and GPT4AD(2) (GPT4AD(2) means using a two-layer GPT-2 when implementing GPT4TS for MTAD tasks).

As demonstrated in Table 3, LGFN attained the optimal average F1-score across the five datasets among all methods. Its recall and accuracy were the second best, and its precision was only 0.88% lower than that of the best-performing method (Autoformer).

The average precision of LGFN was lower than that of Autoformer, GPT4AD, and Anomaly Transformer by 0.88%, 0.87%, and 0.27%, respectively. The following analysis will elucidate the underlying reasons for these observations. First, in terms of the AS calculated in Equation (12), the weighting coefficient *w* for OS is less than 0.5 across all datasets, meaning that it plays a supplementary role in detection. The dominant part is still RS, like in all the other methods, so AS is not significantly reduced in anomalous areas, and TP does not undergo a substantial decrease. Second, in the latent space, the clustering objects are window-level features. In the event of an outlier in a given feature, OS for all time points within that corresponding window is elevated, leading to an increased probability of FP due to normal points near the anomaly being misclassified as anomalies. Upon consideration of both factors, a slight decrease in LGFN’s precision is observed.

LGFN’s average recall ranks second best, only 0.22% lower than the top-performing TimesNet but 4.34% higher than the third-place GPT4AD. In comparison to GPT4AD, LGFN employs a Bi-LSTM encoder–decoder architecture, which has been demonstrated to capture short-term dependencies within a window with greater efficacy than the linear encoder–decoder utilised in GPT4AD. Furthermore, the GPT-2 in LGFN processes a group of time windows’ features as opposed to individual time points, thus allowing it to consider a longer time range. These factors contribute to the model’s enhanced ability to capture both short- and long-term dependencies between time points, thereby rendering it more sensitive to anomalies. Consequently, the effectiveness of the model is enhanced, as evidenced by a reduction in the FN and an increase in recall when compared to GPT4AD. The recall of TimesNet is higher than that of LGFN on the SMD, PSM, and SWaT datasets. This phenomenon can be attributed to the strong periodicity exhibited by these three datasets in many of their dimensions. Specifically, in the SMD dataset, at least 25 out of 38 dimensions display visually discernible periodicity; in the PSM dataset, at least 18 out of 25 dimensions exhibit periodic patterns; and in the SWaT dataset, almost all dimensions manifest clear periodicity. Given that TimesNet incorporates a carefully designed periodic analysis mechanism, its recall outperforms LGFN on datasets with periodicity. As demonstrated in Figure 3, the SMD, PSM, and SWaT datasets manifest more pronounced periodicity in comparison to the MSL and SKAB datasets. In MSL and SKAB, characterised by a weaker periodicity, the relationships between time points and the causes of anomalies are more obscured and profound. In terms of recall, both LGFN and GPT4AD, which use GPT-2, demonstrate superior performance on the two datasets under consideration. It is noteworthy that the SKAB dataset, comprising 11,851 time points, exhibits the highest anomaly rate (28.26%) among the five datasets, thereby establishing it as a substantial test of a model’s capacity to discern patterns and extract features from limited data. Both LGFN and GPT4AD demonstrated particularly strong performance on the SKAB dataset, attaining a recall of approximately 100%, with no missed detections. In the larger-scale MSL dataset, LGFN further distinguishes itself, underscoring the efficacy of the proposed multi-scale feature extraction approach. The GPT-2 in LGFN exhibits a longer learning range than GPT4AD while maintaining reduced spatial complexity.

The F1-score was determined by combining precision and recall. The LGFN model demonstrated the highest average F1-score among the five models, attaining 91.30%, which is a Figure 1.08% higher than the second-best TimesNet model. This outcome suggests that the model effectively balances the two conflicting objectives of precision and recall.

In order to provide a comprehensive evaluation, the accuracy was calculated. Despite LGFN achieving a second-place ranking, it was a mere 0.39 percentage points behind the leading model, GPT4AD. Furthermore, a detailed analysis reveals that LGFN significantly outperforms GPT4AD in terms of space efficiency (see Section 4.3 for a comprehensive analysis).

### 4.3. Memory Usage

It is crucial to emphasize that the aforementioned stages are entirely decoupled from the data undergoing processing. They are designed to operate independently of data size or specific properties; the only restriction is that the input must be a time-continuous and variable amount of usually numerical values. Nevertheless, it is imperative to acknowledge the pivotal role of data volume in determining the efficacy of the AD process. The entire process must be executed efficiently or, at the very least, in a scalable manner, particularly in scenarios where the objective is to cluster fine-grained features (where the window size is relatively small) or to perform AD in small processors with limited computational resources. In order to address this issue, a comprehensive analysis of the memory usage of the proposed LGFN during a single forward pass is provided, thus enabling users to better weigh the feature granularity, detection accuracy, and deployment challenges.

The analysis of LGFN’s structure enabled the calculation of its spatial complexity as

(15) $$\begin{array}{cc} S_{\mathrm{LGFN}}\left(l,M,h,B,S,L,D\right)= & O(\left(l+2\right)hM+2lh+4lh^{2}+2lBSh+\\ \; & 4h^{2}+4h+2hB+BM+LB^{2}D+4LBD), \end{array}$$

where *l* is representative of the LSTM layers contained within ${\mathrm{Bi}-\mathrm{LSTM}}_{\mathrm{enc}}$ and ${\mathrm{Bi}-\mathrm{LSTM}}_{\mathrm{dec}}$, whilst *h* denotes the LSTM hidden size. *B* represents the batch size during a single forward pass, *D* signifies the required input dimension of GPT-2 (which is typically 768), and *L* is indicative of the number of decoder layers in GPT-2.

If the considered sequence length per batch and the embed dimension were set to be the same as LGFN, then the spatial complexity of GPT4AD(*L*) would be

(16) $$S_{\mathrm{GPT}4\mathrm{AD}(L)}\left(M,h,L,B,D\right)=O\left(LBS^{2}D+4LBSD+2hB+BM+2hM\right).$$

The detailed derivation processes for Equations (15) and (16) are provided in Appendix A.

In comparison with GPT4AD, given that ${\mathrm{Bi}-\mathrm{LSTM}}_{\mathrm{enc}}$ extracts window-level features, the sequence length that GPT-2 requires for processing is altered from *T* to T/S. Moreover, given that the self-attention computation within GPT-2 constitutes the primary component of the space utilisation, this reduction in length can diminish the spatial complexity to approximately $B/S^{2}$ of the original. Consequently, the “OUT OF MEMORY” error is effectively alleviated during the AD process.

**Figure 4:**
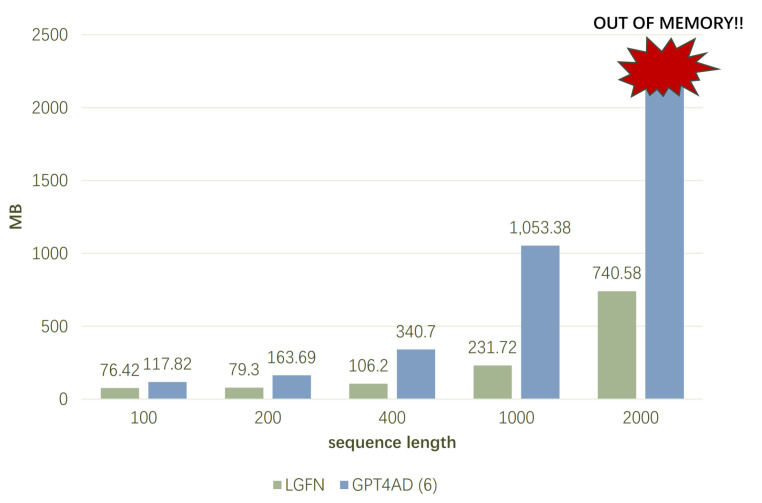
Total CUDA memory used during a single forward pass on the SMD dataset.

Experiments on memory usage are conducted in a hardware environment comprising a 3.00 GHz CPU, 8 GB RAM, 6 GB GPU, and a Windows 10 OS. As illustrated in Figure 4, the total CUDA memory utilised during a single forward pass of LGFN and GPT4AD on the SMD dataset is demonstrated. During the experiments, the variables were controlled, with *L* for both models set to 6, h=300, and other irrelevant parameters set to constant values.

As demonstrated in Figure 4, during the training stage, the CUDA memory usage of LGFN is considerably lower than that of GPT4AD for all sequence lengths. When sequence length T=100, LGFN’s memory usage is 64.86% that of GPT4AD; at a length of 200, this ratio falls below 50%. This decrease in memory usage becomes more pronounced as the sequence length increases, with LGFN’s memory usage being only 22% of GPT4AD’s at a length of 1000. When *T* reaches 2000, GPT4AD encounters an “OUT OF MEMORY” error, whereas LGFN uses only 740.58 MB of GPU space.

### 4.4. Ablation Experiment

To emphasise the significance of each constituent element in the proposed model, each element of the AD process was systematically substituted with more elementary alternatives, and the impact of each replacement on the model’s performance was evaluated.

First, the present study employs the use of RS to evaluate the performance of LGFN, without adding latent space clustering. Second, ${\mathrm{Bi}-\mathrm{LSTM}}_{\mathrm{enc}-\mathrm{dec}}$ is replaced with ${\mathrm{LSTM}}_{\mathrm{enc}-\mathrm{dec}}$, and then a simplest ${\mathrm{Linear}}_{\mathrm{enc}-\mathrm{dec}}$, to extract window-level features. This is undertaken to investigate the impact of the bidirectionality of LSTM and the significance of incorporating short-term time dependencies within a window to enhance AD performance. Third, the multi-GPT-2 layers in LGFN are substituted with a simple transformer decoder layer in order to demonstrate the capability of LLMs and the advantages of pre-training.

The conclusions that can be drawn from Table 4 are as follows:

1. LGFN scores the highest in terms of F1-score, which demonstrates the indispensability of each component within the model.
2. The F1-score, recall and accuracy of the four component replacement methods decrease sequentially from top to bottom, while the FAR increases. This finding suggests that OS, the Bi-LSTM window-level feature extractor, and the GPT-2 batch-level feature extractor incrementally enhance the assistance to AD.
3. Precision undergoes a gradual increase, whilst MAR decreases. This phenomenon can be interpreted as evidence that the incorporation of Bi-LSTM and GPT-2 into the network has led to an enhancement in the network’s capacity to discern anomalies with greater rigour, a property that aligns with human cognitive processes. Furthermore, the minor rise in MAR is predominantly attributable to abnormal time points, which exhibit a comparatively lesser correlation with the machine’s operational health. However, the more critical abnormal time intervals remain predominantly discernible.
4. A comparison of rows 1 and 3 of the table indicates that LGFN, utilising Bi-LSTM as a window encoder, attains a 6.83% higher F1-score than utilising LSTM, highlighting the benefits of bidirectionality in LSTM for AD. This suggests that anomalies in time series data have two-way local dependencies within a window, meaning the points immediately before and after an anomaly are also likely to be anomalous. In practical terms, this can be interpreted as a machine malfunctioning at time *t* possibly being caused by a faulty state before *t*, which could also affect its performance after *t*.
5. Comparing rows 3 and 4 of the table, replacing LSTM with a simpler linear encoder–decoder structure results in a further decrease in F1-score, confirming that LSTM, which can account for short-term dependencies, is more effective than encoding structures that cannot.
6. The last row of the table shows that replacing GPT-2 with a single-layer transformer encoder–decoder results in the largest drop in F1-score (down 26.97%), emphatically demonstrating the capability of large models and the importance of pre-training, which is the most significant aid to AD.

### 4.5. Experiments on the Sensitivity of *w*

**Figure 5:**
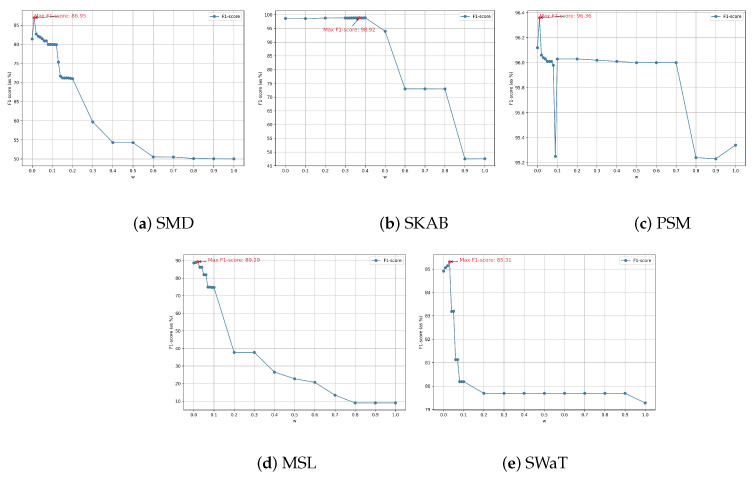
F1-score sensitivity to *w* on five datasets.

The weighting parameter *w* in Equation (12) is undoubtedly crucial to the model’s AD performance. To provide a method for pre-selecting *w* on a new dataset, we conducted a hyperparameter sensitivity analysis on *w* and plotted the variation of the F1-score as *w* fluctuates within the range [0, 1], as shown in Figure 5. This can help justify its optimal setting.

The value of *w* is changed from 0 to 1 with a step size of 0.1. Once the range where the maximum F1-score occurred is roughly identified, the step size is further reduced to 0.01 to compute a more precise *w* value where the maximum occurred. As *w* changes from 1 to 0, we observe two common trends in the F1-score across the five datasets: First, the F1-score reaches its maximum value when *w* is in the range (0, 0.4); second, as *w* continues to increase, the F1-score drops sharply. This indicates that RS remains the main component for ensuring detection accuracy, while OS can improve detection accuracy but only plays an auxiliary role. A closer analysis of the *w* values where the maximum F1-score occurs shows that for Figure 5a,c,d,e, the optimal *w* falls within the range [0.0, 0.1]. Only Figure 5b has its *w* in the range [0.3, 0.4], but its change in F1-score across the range [0, 0.4] is relatively small, and the *w* value in this range is nearly identical to the maximum value. This suggests that for a new dataset, setting *w* within the range [0.0, 0.1] can improve model detection accuracy.

The auxiliary role of *w* is more prominent in Figure 5a, the SMD dataset, where the highest F1-score of 86.95% occurs at w=0.01, which is a local maximum in its neighbourhood. The F1-scores at w=0.00 and w=0.02 are 81.40% and 82.69%, respectively, differing from the maximum by 5.55% and 4.26%. In Figure 5c PSM, although a relatively large drop occurs at w=0.09, the difference from the maximum F1-score does not exceed 1.2%. It should be noted that, under the step size conditions for *w* in this experiment, the F1-score change curve is overall smooth, which is why no further reduction in the step size was made. As for the reasons behind the occurrence of the extreme values and the selection of more suitable clustering methods in the OS calculation, further investigation is needed in future research.

### 4.6. Anomaly Score and Latent Space Analysis

During the AD process, both normal and abnormal samples are input into LGFN. After two-scale learning, outliers corresponding to abnormal windows will emerge and be detected in the latent space using OS. Moreover, ${\mathrm{Bi}-\mathrm{LSTM}}_{\mathrm{dec}}$ will struggle to accurately conduct reconstruction using outlier feature vectors. Therefore, RS at abnormal time points will be higher than at normal time points. In order to facilitate an intuitive and human-friendly understanding of the process by which OS detects anomalies in latent space, the use of visualisation plots of the clustering results is recommended. These plots can also be used to indicate the minimum number of clusters required to effectively separate the features prior to any clustering operation [42].

**Figure 6:**
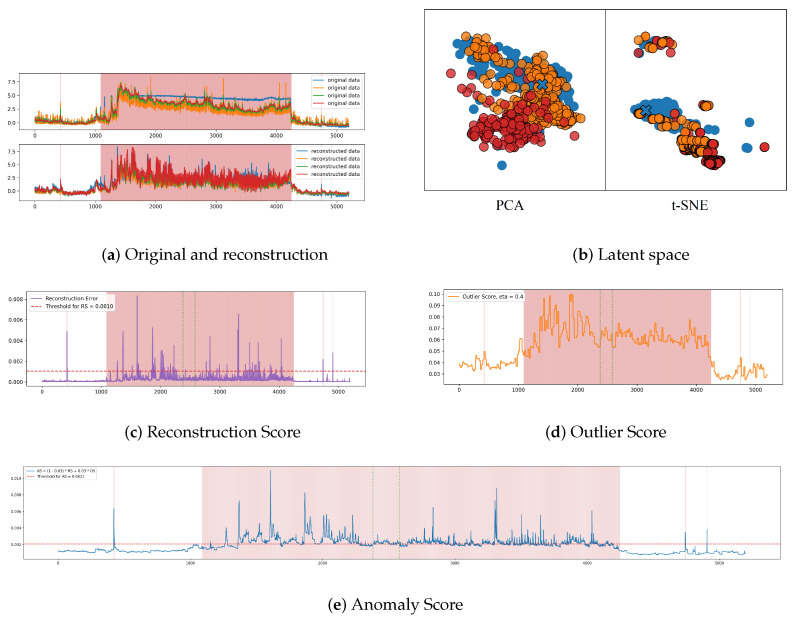
Scores and latent space visualisations. (**a**) Original data (top) and reconstructed data (bottom) from a segment of the SMD test set. Due to space limitations, only four dimensions are shown. (**b**) Latent space features of the windows in (**a**), reduced to 2D using PCA (left) and t-SNE (right). Blue points represent normal features from the training set (known class), orange points represent normal features from the test set, and red points represent anomalous features from the test set. The blue cross is the class centre of all these features, which is used to calculate the deviation degree of each feature from the test set in this cluster. (**c**) RS for the data segment in (**a**), with the red dashed line indicating the threshold for AD based solely on RS. (**d**) OS for the data segment in (**a**). (**e**) AS obtained by weighting RS and OS, with the red dashed line representing the threshold for AD using AS, which is the final detection result. The red shading indicates the anomalous time points (segments) in all figures.

To illustrate this, Figure 6a shows a comparison between an input sub-sequence (top) from the SMD test set and their reconstructed counterparts (bottom). The figure demonstrates that the reconstructions for normal samples closely match the original data. However, when the input contains anomalies, indicated by the red shading in the figure, the reconstructed sequences fail to replicate these abnormal points. This discrepancy facilitates AD in the original input space, as shown in Figure 6c.

In Figure 6b, the 2D latent space following PCA and t-SNE dimensionality reduction for the features corresponding to the subsequences depicted in Figure 6a is shown. Orange points represent normal features, while red points denote anomalous features, both originating from the test set. The blue points correspond to features from the training set that do not contain anomalies, with blue crosses indicating the class centres of these training features obtained via k-medoids clustering. It is evident from the figure that, although both the normal and anomalous test features are assigned to the same class represented by the blue crosses, most of the red points are farther from the class centre than the orange points. This indicates that the anomalous features exhibit a higher deviation degree, which aids in AD in the latent space. It is evident that the distribution of 2D feature vectors can be influenced by dimensionality reduction methods, as demonstrated in Figure 6b. The 2D latent space visualisations of feature vectors from the same cluster obtained using PCA and t-SNE will differ. Consequently, the 2D feature distribution should be regarded as a reference only, while OS in Figure 6d provides a clearer representation of distances from different windows to their respective class centres.

As illustrated in Figure 6c, the area delineated by the two green dashed lines signifies FNs when employing solely RS for detection. Conversely, the corresponding segment in (b) exhibits a higher OS, suggesting that the features of this segment are more distant from the centre of their class in the latent space. Consequently, in (e), the weighted average of both scores exceeds the threshold, accurately identifying these points as anomalies and categorising them as TP. This finding underscores the efficacy of OS in enhancing RS’s capacity for precise detection.

**Figure 7:**
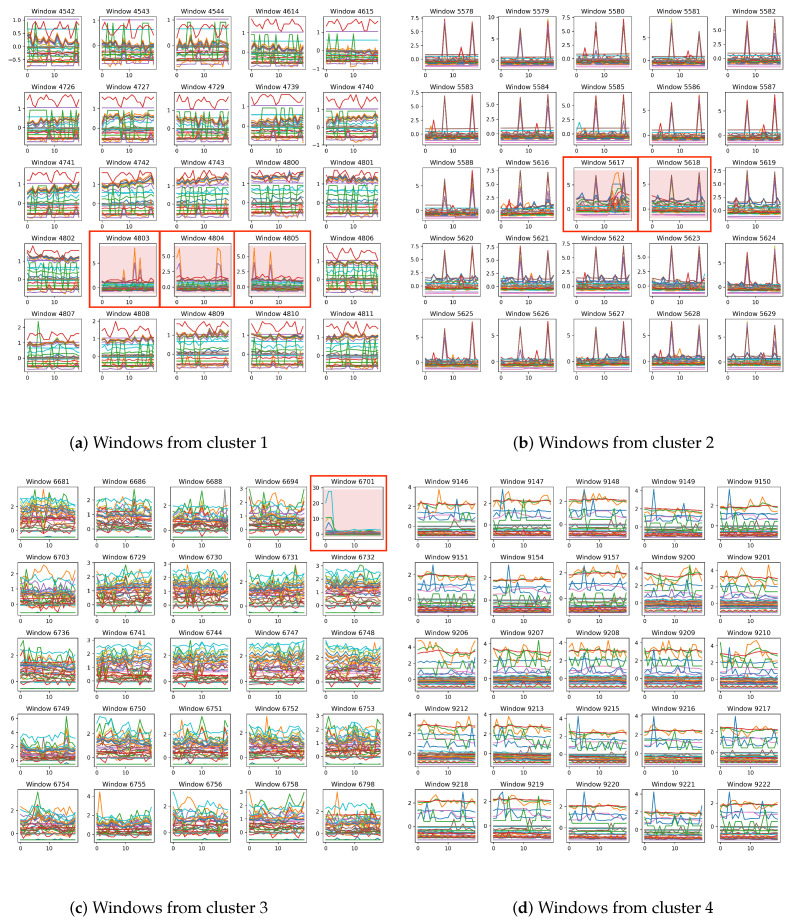
SMD test set windows from four different clusters.

Figure 7 shows windows corresponding to parts of features from four different clusters of the SMD test set, with anomalous time points highlighted in red and enclosed with red boxes. In one window, different MTS channels are distinguished using different colours. From this figure, the following two phenomena can be summarized:

1. Most of the windows assigned to the same cluster come from adjacent periods; even windows separated by some time intervals exhibit certain similarities in trends and states. Other windows in this subplot, such as 4544 in the first row, third column, and 4614 in the first row, fourth column, although not continuous in time, exhibit strong similarity. Specifically, the features corresponding to these two windows are very close in the latent space, which is why they are grouped into the same class.
2. Windows belonging to the same cluster exhibit clear visual similarities, while the differences between anomalous and normal windows within the same cluster are significant. For example, the windows in Figure 7b share a common and obvious characteristic: two peaks appear at the 7th and 17th time steps across multiple dimensions, while the other dimensions remain relatively smooth. However, the two anomalous windows, 5617 and 5618, exhibit different characteristics: in window 5617, at least two dimensions show anomalous peaks at the 15th time step; in window 5618, a particular red dimension shows a significant decrease in value. These unique behaviours lead to their features being outliers in the latent space.

These two phenomena visually demonstrate the effectiveness of clustering-based pattern mining in latent space, and they further suggest that OS is indeed helpful to AD.

## 5. Conclusions and Future Work

In this paper, a model called the LSTM-GPT2 Fusion Network (LGFN) is proposed for solving MTAD tasks. The model provides an effective solution to address the limitations of existing AD methods, including a lack of robustness to noisy data, inability to effectively capture long-term temporal relationships, insufficient utilization of latent space features, and challenges in deployment on small-scale devices.

The model has two functions: multi-scale feature extraction and data reconstruction. AD using LGFN can be divided into three stages: the training stage, the testing stage, and the anomaly detection stage. In the training stage, the model is forced to learn normal behaviours using reconstruction error between the original input and the network output. After training, ${\hat{Z}}_{\mathrm{train}}$ are collected for the later AD stage. In the testing stage, the test set interoperates both normal and anomalous data, generated under the same operating conditions as the training set. ${\hat{Z}}_{\mathrm{test}}$ and ${\hat{X}}_{\mathrm{test}}$ are collected as well. In the anomaly detection stage, RS is calculated using $X_{\mathrm{test}}$ and ${\hat{X}}_{\mathrm{test}}$. Pattern mining is conducted by latent space clustering on ${\hat{Z}}_{\mathrm{train}}$. The obtained centres represent standard working patterns. ${\hat{Z}}_{\mathrm{test}}$ is then distributed to these known clusters. Using the distribution results, OS is calculated based on the distance between each feature in ${\hat{Z}}_{\mathrm{test}}$ and its centre. Next, by using the hyperparameter *w* to weight and sum these two scores, an enhanced AS is obtained. Finally, a percentile-based threshold is applied to produce a binary output ${\hat{y}}_{t}\in \left\{0,1\right\}$ for each data point in the test set. Metrics of AD can be calculated using the model prediction ${\hat{Y}}_{\mathrm{test}}$ and the test set ground truth $Y_{\mathrm{test}}$.

Upon comparison with five advanced AD methods under five widely recognised MTS datasets, it can be seen that utilising LGFN to perform AD can achieve state-of-the-art performance or comparable results. In memory usage analysis, the spatial complexity of LGFN is theoretically calculated, and experiments demonstrate that the GPU usage of LGFN is much lower than that of GPT-2 when processing raw time series data, thus alleviating the “OUT OF MEMORY” error that often occurs during the training of large models. In the context of ablation studies, all primary components are systematically removed or replaced, with a subsequent decline in most metrics indicating the indispensability of each module in the proposed AD process. The final experiment section provides a latent space visualisation, elucidating the manner in which OS can assist RS in achieving a more accurate detection.

Despite the efficacy of the proposed methodology in accurately and expeditiously detecting anomalies in time series, it is important to acknowledge the limitations inherent to the approach. One such limitation pertains to the improvement in AD efficiency, which results in a marginal decline in precision. Another is the requirement of datasets from OS calculation. Specifically, the data employed for network training and testing must be sourced from the same operational environment. To illustrate, if the training data consist of vibration signals from Engine No.1 during normal operation, captured by 11 different sensors (M=11, with all labels being 0), then the test data must also be vibration signals from the same 11 sensors, capturing the machine’s behaviour during both normal and abnormal operations (*M* must remain 11, with alternating 0/1 labels). These 11 sensors can be deployed on different machines, but it is essential that the machines are of the same type (for example, all engines, such as Engine No. 2, No. 3, etc., but not hydraulic presses or CNC machines). Ideally, the sensor placement should also be consistent across machines. In the event of non-consistency, the entirety of the features from the designated test set would be classified as outliers, consequently resulting in a high deviation degree across all metrics.

The present study acknowledges the potential for further development in this area of research. In the present state, the method utilises a static threshold of AS. This approach is limited in its capacity to demonstrate the advanced AD capabilities of the proposed model. In order to address this limitation, it is necessary to employ a well-designed method to obtain a dynamic threshold that can adapt to the fluctuations in AS. This would allow the performance of LGFN to be enhanced, with all metrics surpassing those of state-of-the-art methods.

## Figures and Tables

**Table 1:** Experimental environments.

Component	Specification
Operating System	Windows 10 Professional
CPU	Intel(R) Core(TM) i7-9700 CPU @ 3.00 GHz
GPU	NVIDIA GeForce 2060
Memory	8 GB
GPU Memory	6 GB
Python Version	3.11.9
PyTorch Version	2.3.1+cu121

**Table 2:** Dataset Information and Experimental Settings.

Datasets	SMD	SKAB	PSM	MSL	SWaT
***M***	38	8	25	55	51
** $T_{\mathrm{train}}$ **	194,374	11,851	132,481	58,317	495,000
** $T_{\mathrm{test}}$ **	194,369	4312	87,841	73,729	449,919
** $T_{\mathrm{train}}:T_{\mathrm{test}}$ **	1:1	3:1	3:2	4:5	1:1
**Anomaly Rate**	4.16%	28.26%	27.69%	10.72%	12.19%

**Table 3:** Method comparison on anomaly detection tasks. F1-score = 2 · (precision · recall)/(precision + recall), precision = TP/(TP + FP), recall = TP/(TP + FN), and accuracy = (TP + TN)/(TP + TN + FP + FN) (as %) are calculated for each dataset. Red: best, **Black**: second best.

Metrics	Methods	SMD	SKAB	PSM	MSL	SWaT	Average
**F1-score**	**LGFN (Ours)**	**86.95**	**98.92**	96.39	89.29	**85.31**	91.30
	**GPT4AD(2)**	81.32	99.16	97.89	85.86	80.15	88.88
	**TimesNet**	86.33	94.44	**97.33**	80.38	92.60	**90.22**
	**Autoformer**	86.92	91.55	88.85	81.03	79.19	87.51
	Anomaly *	88.72	83.25	91.99	**88.49**	81.05	86.70
	**LSTM-VAE**	80.65	66.04	70.17	80.36	79.95	75.43
**Precision**	**LGFN (Ours)**	90.07	97.87	**99.35**	92.70	99.17	95.83
	**GPT4AD(2)**	97.98	98.34	96.05	91.48	**99.66**	**96.70**
	**TimesNet**	86.15	**98.56**	98.39	88.69	92.10	92.78
	**Autoformer**	**93.94**	99.32	99.99	90.34	99.96	96.71
	Anomaly *	92.38	97.57	99.21	**91.73**	99.62	96.10
	**LSTM-VAE**	91.35	59.80	70.25	90.40	93.76	81.11
**Recall**	**LGFN (Ours)**	84.03	100.0	93.59	86.11	**74.85**	**87.79**
	**GPT4AD(2)**	69.50	100.0	99.80	80.89	67.04	83.45
	**TimesNet**	86.51	90.65	**96.29**	73.50	93.11	88.01
	**Autoformer**	80.88	**94.91**	79.94	73.74	65.57	79.00
	Anomaly *	**85.33**	72.59	85.76	**85.47**	68.31	79.49
	**LSTM-VAE**	72.19	73.73	70.09	72.33	69.69	71.61
**Accuracy**	**LGFN (Ours)**	98.47	**99.32**	98.06	97.83	**96.86**	**97.51**
	**GPT4AD(2)**	98.07	99.48	98.81	97.19	95.97	97.90
	**TimseNet**	98.34	96.24	**98.53**	96.22	98.19	97.50
	**Autoformer**	**98.52**	94.49	94.43	96.37	95.82	95.93
	Anomaly *	98.69	89.98	95.86	**97.65**	96.12	95.66
	**LSTM-VAE**	97.90	76.48	83.50	96.27	95.74	89.98

* means Transformer. The results of GPT4AD(2) were reproduced by [40]. The results of TimesNet and Autoformer were reproduced by [41].

**Table 4:** Metrics of the ablation experiment on the SMD dataset (as %). FAR = FP/(FP + TN) is the false alarm rate (the lower the FAR, the better), and MAR = FN/(FN + TP) is the missed alarm rate (the lower the MAR, the better). The lower these two metrics are, the better the model’s AD performance. Red: best, **Black**: second best.

Methods	F1-score	Precision	Recall	Accuracy	FAR ↓	MAR ↓
LGFN	86.95	90.70	84.03	98.47	1.023	5.977
- AS –> RS	**81.39**	98.14	**69.53**	**98.08**	**1.927**	1.863
- Bi-LSTM –> LSTM	80.12	98.84	67.37	97.98	2.060	1.162
- Bi-LSTM –> Linear	76.81	**99.07**	62.72	97.71	2.346	**0.928**
- GPT-2 –> Transformer	59.98	99.76	42.88	96.54	3.550	0.238

## Data Availability

Data are contained within the article.

## Abbreviations

The following abbreviations are used in this manuscript:

AD	Anomaly Detection
MTS	Multivariate Time Series data
MTAD	Multivariate Time series Anomaly Detection
EWMA	Exponential Weighted Moving Average
ML	Machine Learning
DL	Deep Learning
RNN	Recurrent Neural Network
LSTM	Long Short-term Memory
Bi-LSTM	Bi-directional LSTM
LLM	Large Language Model
NLP	Natural Language Processing
GPT	Generative Pre-trained Transformer
GAN	Generative Adversarial Network
CNN	Convolutional Neural Network
LGFN	LSTM-GPT2 Fusion Network
MSE	Mean Square Error
GPT4AD	GPT-2 for Time Series Anomaly Detection tasks
RS	Reconstruction Score
OS	Outlier Score
AS	Anomaly Score

## Appendix A. Analysis of Spatial Complexity

The layers inside LGFN are: ${\mathrm{Bi}-\mathrm{LSTM}}_{\mathrm{enc}}$, ${\mathrm{Bi}-\mathrm{LSTM}}_{\mathrm{dec}}$, GPT-2, and two fully connected layers. The encoder-decoder architecture’s spatial complexity is

(A1) $$\begin{array}{cc} S_{e-d}\left(l,M,h,B,S\right) & =S_{\mathrm{encoder}}\left(l,M,h,B,S\right)+S_{\mathrm{decoder}}\left(l,h,B,S\right)\\ \; & =O\left(lhM+lh^{2}+lh+lBSh\right)+O\left(3lh^{2}+lh+lBSh\right)\\ \; & =O\left(lhM+4lh^{2}+2lh+2lBSh\right). \end{array}$$

The GPT-2 layers inside LGFN process the features of all time windows within a batch, so for GPT-2, the sequence length is equal to the batch size *B*, and the batch size equals 1. The corresponding spatial complexity is

(A2) $$S_{\mathrm{gpt}-2}\left(L,B,D\right)=O\left(LB^{2}D+4LBD\right).$$

Besides the above two dominant parts, two linear layers are used: one for feature transformation after GPT-2, where the input and output dimensions are both 2H; the other is applied after the decoder, mapping the embedding dimension 2h to the original data dimension *M*.

(A3) $$\begin{array}{cc} S_{\mathrm{linear}}\left(h,M,B\right) & =S_{\mathrm{linear}-1}\left(h\right)+S_{\mathrm{linear}-2}\left(h,M,B\right)\\ \; & =O\left(4h^{2}+4h\right)+O\left(2hM+2hB+BM\right)\\ \; & =O\left(4h^{2}+4h+2hM+2hB+BM\right). \end{array}$$

Adding the three parts shown in Equations (A1)–(A3), the total spatial complexity of LGFN in Equation (15) can be obtained.

The GPT4AD(*L*) is comprised of two principal components: GPT-2 with *L* transformer decoders and a linear projection layer. In order to facilitate a more effective comparison of the spatial efficiency of the two models by controlling variables, it is necessary to set the length of the time series data processed by GPT-2 inside GPT4AD(*L*) in one batch equals B×S, and the embedding dimension to be 2h, both of which are the same as LGFN. It is evident that these transformer layers constitute the predominant component of $S_{\mathrm{GPT}4\mathrm{AD}(L)}\left(M,h,L,B,D\right)$ in Equation (16), calculated as follows.

(A4) $$\begin{array}{cc} S_{\mathrm{GPT}4\mathrm{AD}(L)}\left(M,h,L,B,D\right) & =S_{\mathrm{gpt}-2}\left(L,B,S,D\right)+S_{\mathrm{linear}}\left(h,M,B\right)\\ \; & =O\left(LBS^{2}D+4LBSD\right)+O\left(2hB+BM+2hM\right)\\ \; & =O\left(LBS^{2}D+4LBSD+2hB+BM+2hM\right). \end{array}$$
